# Principles and guidelines in the management of ankle fractures in adults

**DOI:** 10.1177/1750458920969029

**Published:** 2021-04-07

**Authors:** Harry Kyriacou, Ahmed M H A M Mostafa, Benjamin M Davies, Wasim S Khan

**Affiliations:** 1 School of Clinical Medicine, University of Cambridge, Cambridge, UK; 2 Department of Trauma & Orthopaedics, Addenbrooke's Hospital, Cambridge, UK

**Keywords:** Ankle fracture, Perioperative, Complications, Orthopaedics

## Abstract

Ankle fractures are common injuries that have many physical and psychosocial complications. As a result, it is important to be aware of how these patients present and are managed perioperatively. Detailed guidelines from NICE and the British Orthopaedic Association have been produced on this topic, including recent developments such as the decision to weight-bear early after surgery and the use of virtual fracture clinics. This article provides an overview of the key perioperative factors that need to be considered in cases of ankle fracture and the relevant clinical guidelines.

**Provenance and Peer review:** Unsolicited contribution; Peer reviewed; Accepted for publication 6 Oct 2020.

## Introduction

Ankle fractures are common, representing 14% of all fractures requiring hospitalisation (Jennison & Brinsden 2019). Between 2004–05 and 2013–14, there were 332,617 hospital admissions in England due to ankle fractures, accounting for 10% of hospital bed stays (Jennison & Brinsden 2019). Ankle fractures affect the lateral malleolus in 55% of cases and commonly occur due to sports injuries in adolescents (22%) or low-energy falls in later years (61%) (Elsoe et al 2018). They are associated with significant morbidity in all age groups and have a one-year mortality rate of 11.9% after hospitalisation in patients over the age of 65 years (Hsu et al 2015).

The National Institute for Health and Care Excellence (NICE) has published detailed guidelines on how to assess, monitor and manage ankle fractures (NICE 2016a, 2016b). Partly based on these guidelines, the British Orthopaedic Association (BOA) has published standards for practice in the management of ankle fractures, known as the British Orthopaedic Association Standards for Trauma and Orthopaedics (BOAST) guidelines (BOA 2016). This article provides an overview of ankle fractures and their relevant clinical guidelines and presents them in a readable format that follows the patient journey. The aim of this article is to raise awareness of these injuries, particularly in the perioperative period, to optimise the care that these patients receive.

## Clinical anatomy

**Figure 1:**
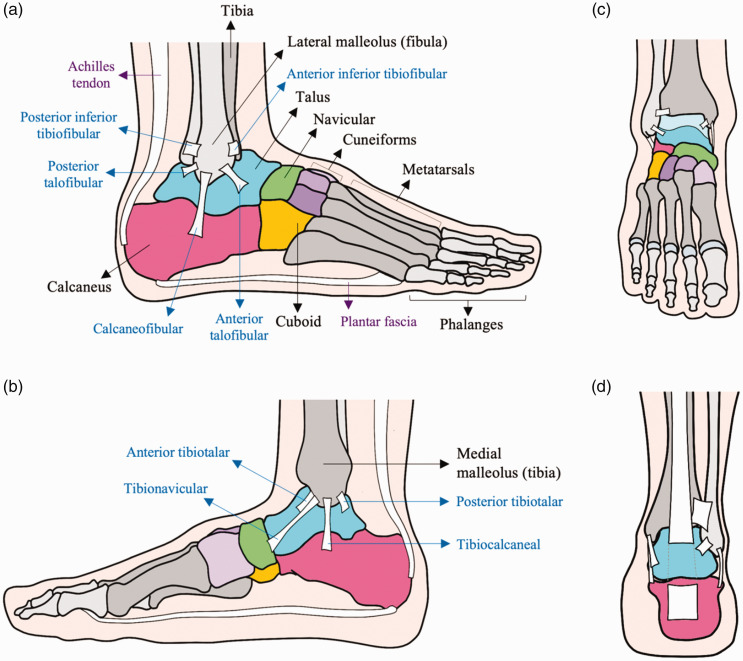
Anatomy of the ankle. Right foot in various views: (a) lateral; (b) medial; (c) anterior; (d) posterior (a part of the Achilles tendon has been removed). The names of the corresponding ligaments are given in blue.

The ankle joint is a synovial mortise and tenon made up of the articular surface of the tibia, both malleoli and the talus (Moore et al 2014). It works with the subtalar joint to act as a modified hinge which can plantar-flex, dorsi-flex, glide and roll (McKeon & Hoch 2019). To add stability, the ankle is also bound by three lateral ligaments and a strong medial deltoid ligament (Moore et al 2014). This is therefore a complex region with many potential sites of injury (Figure 1).

## Preoperative considerations

### Presentation, history and examination

Ankle fracture patients typically present with immediate-onset ankle pain that increases with activity, localised or generalised swelling, bruising, joint deformity and an inability to weight-bear (Slimmon & Brukner 2010). Severe ankle sprains may present with similar features. Therefore, history-taking and examination are particularly important in distinguishing between different types of ankle injury.

In the history, it is important to ascertain the timing of injury, mechanism (inversion, eversion, plantar flexion or dorsiflexion), severity (force and velocity involved) and whether the patient was able to bear weight immediately after. The presence and onset of ankle swelling also need to be established, as acute-onset swelling may suggest bleeding (Slimmon & Brukner 2010). A past medical history should include previous ankle injuries which may have predisposed the joint to fracture (Slimmon & Brukner 2010). In addition, comorbidities that might influence treatment choice and outcome should be documented, eg pre-existing mobility impairment, diabetes mellitus, peripheral neuropathy, peripheral vascular disease, osteoporosis, renal disease, smoking and alcohol overuse (BOA 2016).

Examination should include inspection for deformity, bruising, effusion and open wounds (BOA & BAPRAS 2017; Lampridis et al 2018). Palpation is performed in a methodical sequence across both the lateral and medial ankle as well as proximal fibula (for a potential Maisonneuve injury) (Lampridis et al 2018). Tenderness is suggestive of an underlying fracture rather than sprain. A neurovascular assessment of the foot should also be performed compared with the contralateral limb, and the results documented (NICE 2016b). This includes sensation over the dorsal and plantar surfaces of the foot, distal pulses and capillary refill in all digits (Mordecai & Al-Hadithy 2011).

### Initial management

The latest NICE guidelines emphasise the importance of regularly assessing pain in ankle fracture patients, using a scale suitable for the patient’s age, developmental stage and cognitive function (NICE 2016a). Oral paracetamol should be offered for mild pain, oral paracetamol and codeine for moderate pain and intravenous (IV) paracetamol with IV morphine titrated to effect for severe pain (NICE 2016a). IV opioids should be used with caution in elderly patients, and non-steroidal anti-inflammatory drugs should be avoided (NICE 2016a).

Clinically deformed ankles require urgent reduction and splinting (BOA 2016). Radiographs should not be performed before reduction if they will cause an unacceptable delay (BOA 2016). Reduction minimises the risk of skin necrosis and reduces pain and swelling (Mordecai & Al-Hadithy 2011). After reduction, the neurovascular status should be reassessed and documented (BOA 2016). Any fracture should be stabilised in a well-fitted backslab cast or splint, with the limb elevated and a post-reduction X-ray arranged to confirm adequate alignment (BOA 2016; Mordecai & Al-Hadithy 2011).

Patients with open fractures, which make up 1.5% of all ankle fractures, should have any gross contaminants removed and their injury photographed (BOA & BAPRAS 2017; Bugler et al 2015). The fracture site should then be covered with a saline-soaked sterile dressing and wrapped loosely with an occlusive film whilst awaiting debridement surgery (BOA & BAPRAS 2017). Intravenous antibiotic prophylaxis should be given as soon as possible, preferably within 1 hour of injury (BOA & BAPRAS 2017). Debridement should occur immediately for highly contaminated open fractures, within 12 hours for high-energy open fractures that are not highly contaminated and within 24 hours for all other open fractures (BOA & BAPRAS 2017). Fixation and definitive soft tissue cover should be performed at the same time where possible, or if not possible, within 72 hours of injury (NICE 2016b).

Following initial management, the ankle should be immobilised using a splint. Then, definitive treatment is chosen depending on the stability of the joint (Lampridis et al 2018). Patients with stable fractures or co‐morbidities rendering them unfit for surgery are treated conservatively with analgesia and immobilisation using a splint, short‐leg cast or walker boot for a minimum of six weeks (Lampridis et al 2018; Moredecai et al 2011). In addition to this, the BOA recommends close contact casts for patients over 60 years of age as an alternative to surgery if reduction can be maintained by the cast (BOA 2016). These patients exhibit poor bone and soft tissue quality which leads to poor surgical outcomes (Srinivasan & Moran 2001). By comparison, unstable ankle fractures are generally treated surgically as discussed later. This may be preceded by temporary external fixation to allow soft tissue swelling to settle down in some cases, eg high‐energy fractures of the distal tibial articular surface (known as pilon fractures) (Shah et al 2019).

### Imaging

It can often be difficult to clinically distinguish between bony and ligamentous ankle injuries. Historically, this meant that most patients would receive costly, time-consuming X-rays and unnecessary radiation exposure (Stiell et al 1992). As a result, the ankle Ottawa rules were created to guide which patients needed to be imaged. These rules state that an X-ray is only required if there is malleolar pain and any one of the following: an inability to weight-bear immediately and in the emergency department for four steps; bony tenderness of the posterior tibia/fibula or bony tenderness at the tips of the medial/lateral malleolus (Stiell et al 1992). The literature suggests that the Ottawa ankle rules have a sensitivity of almost 100%, with a specificity of 39.8%, and have reduced unnecessary radiographs by 30–40% (Bachmann et al 2003).

Imaging should include lateral and mortise view radiographs (Vangsness et al 1994). A mortise view is an anteroposterior image of the leg in 15–20° of internal rotation. If radiographs are equivocal or significant ligament/tendon injury is expected, a magnetic resonance imaging scan is the imaging of choice (Sawant & Sanghvi 2018). Computed tomography (CT) scanning is useful for the assessment of complex ankle fractures, especially those involving the posterior malleolus and/or comminuted malleolar fractures (Lampridis et al 2018; Leung et al 2016).

### Classification

**Figure 2:**
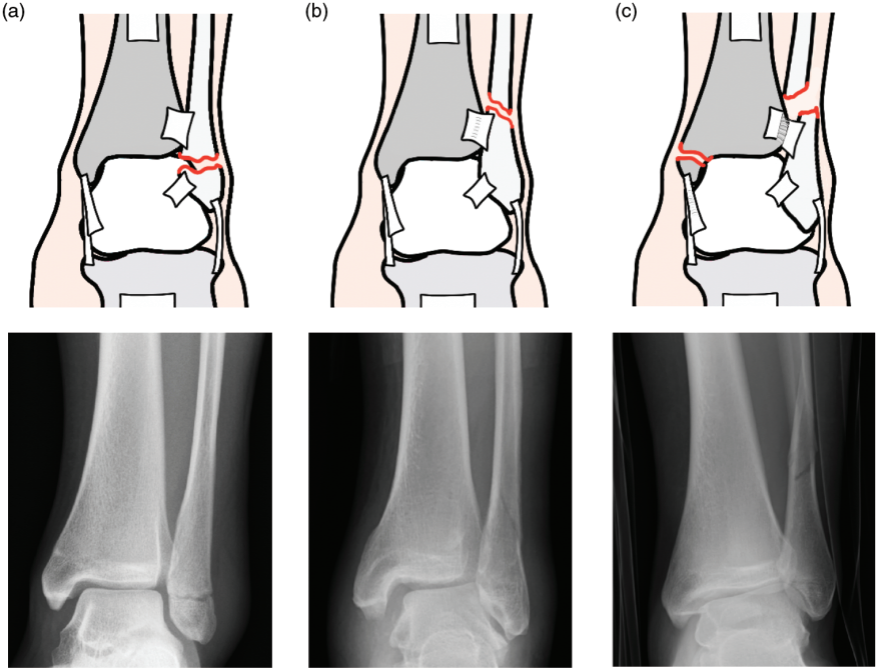
The Danis–Weber classification system. Right foot viewed posteriorly: (a) Type A; (b) Type B; (c) Type C with disrupted syndesmosis.

Ankle fractures can be classified using the Danis–Weber classification system (shown in Figure 2). Type A fractures involve the lateral malleolus distal to the tibiofibular syndesmosis. They are usually stable and are managed conservatively (Donken et al 2012). Type B fractures occur at the level of the syndesmosis and have variable stability. They can be treated both conservatively and surgically (Donken et al 2012). Type C fractures occur proximal to the syndesmosis, which is often disrupted, and are unstable. In these cases, there is usually a concurrent fracture of the medial malleolus or injury to the deltoid ligament. Type C fractures are likely to require open reduction and internal fixation (ORIF) (Donken et al 2012).

Other less commonly used ankle fracture classifications include the Lauge–Hansen classification which focuses on the mechanism of injury, as determined by a series of cadaver-based experiments (Lauge-Hansen 1954). The classification of ankle fractures is a dynamic topic, and new schemes have been proposed as recently as 2019 (Briet et al 2019).

## Operative considerations

### The decision to operate

Unstable fractures are treated surgically in patients deemed fit enough to undergo surgery. These generally include: fractures with syndesmotic disruption (Weber type C and some type B fractures); displaced fractures; unstable bi-/tri-malleolar fractures and fractures with joint incongruity or talar subluxation (Mordecai & Al-Hadithy 2011). The decision to treat posterior malleolar fractures is more controversial, with some authors recommending surgical fixation for fracture fragments that range from 10 to 25% of the distal tibial articular surface (Odak et al 2016). To evaluate fragment size, many surgeons now advocate obtaining a CT scan of the ankle in all patients with a known or suspected posterior malleolus fracture, although not yet part of British national guidelines (Solan & Sakellariou 2017). Recently, there has been a move towards fixation of more posterior malleolar fractures, as even with small areas of the articular surface, there can be significant disruption to the posterior components of the syndesmosis (Bartoníček et al 2017). NICE emphasises the importance of early fixation by advising surgery on the day of injury or the day after in patients under 60 years of age (NICE 2016a).

### Choice of surgery

**Figure 3:**
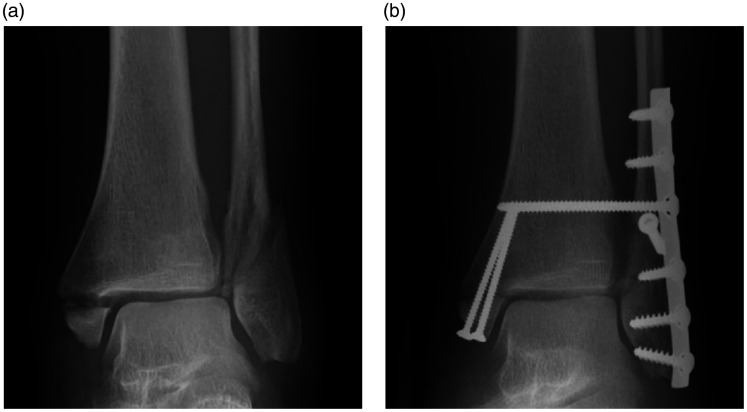
Open reduction and internal fixation. Mortise view of an unstable lateral and medial malleolar ankle fracture before surgery (a) and after ORIF (b).

Surgical treatment mostly takes the form of ORIF using plates and screws to reduce and stabilise the mortise (BOA 2016) (Figure 3). Intraoperative fluoroscopy is used to monitor reduction and fixation (BOA 2016). Lateral malleolar fractures are most commonly fixed using an interfragmentary lag screw and a neutralisation plate (Lampridis et al 2018). However, if the fracture pattern allows more than one lag screw, a neutralisation plate is not mandatory (Lampridis et al 2018). Comminuted high-energy fibula fractures require stronger fixation with locking or reconstruction plates (Lampridis et al 2018). Alternatively, unstable distal fibular fractures can be treated using intramedullary fixation. This is a minimally invasive technique that has been shown to produce results comparable with plating, including a mean rate of union of 98.5% (Jain et al 2014).

Medial malleolar fractures, by comparison, are commonly fixed using lag screws or a tension band wire if the fracture fragment is small (Lampridis et al 2018). For comminuted medial malleolar fractures, smaller-sized screws are recommended. Another method used to treat vertical medial malleolar fractures specifically involves the application of a buttress plate or lag screws in combination with a plate (Lampridis et al 2018). Traditionally, posterior malleolar fractures have been stabilised using anteroposterior screw fixation, although many posterior malleolar fractures are managed nonoperatively in conjunction with surgically treated lateral or medial fractures (Odak et al 2016). Stabilising lateral or medial fractures inherently improves posterior fragment stability in these patients. More recently, there has been a trend towards fixation of posterior malleolus fractures using postero-lateral and postero-medial approaches which allow for more biomechanically sound fixation of the posterior malleolus (Gandham et al 2020).

After the ankle mortise is fixed, the syndesmosis must be assessed intraoperatively using a stress test such as the Hook test. This involves applying a lateral force to the distal fibula in order to obtain a radiograph of the mortise under stress (Stoffel et al 2009). If instability is identified, syndesmotic stabilisation is required. A number of options exist for stabilising the syndesmosis including screw fixation, with variation in the number of screws used and cortices crossed as well as the use of TightRope® system. This system offers an alternative to conventional screw fixation that doesn’t require removal and allows some movement at the syndesmosis. However, there is no clear evidence that one method is superior to another (Lampridis et al 2018).

## Postoperative considerations

### Physical and psychosocial complications

After surgery, patients are followed up within six weeks to assess the stability and alignment of the ankle and to monitor for any surgical complications (BOA 2016). It is especially important to be vigilant for postoperative complications amongst the elderly due to a high incidence of comorbidities and poor bone quality (Srinivasan & Moran 2001). Complications may include deep vein thrombosis and pulmonary embolism secondary to postoperative immobilisation and casting (Mehta et al 2014). Thromboprophylaxis should therefore be initiated in accordance with local guidelines, and early mobilisation should be considered where appropriate as discussed below (BOA 2016).

Deep postoperative infection is a potentially limb-threatening complication that occurs in 3.4% of ankle fractures (Macera et al 2018). A high index of suspicion is, therefore, necessary in order to facilitate early recognition and treatment. Management includes broad-spectrum IV antibiotics and debridement surgery (Zalavras et al 2009). Diabetic patients need careful perioperative monitoring as they are particularly prone to infection. A recent study reported that the rate of infection in operatively treated diabetic ankle fracture patients varied from 11.5 to 21.5%, with higher rates in type 1 compared with type 2 diabetes and in open compared with closed fractures (Haddix et al 2018). Diabetic patients are also at an increased risk of other complications including impaired wound healing, malunion, non-union, Charcot arthropathy and soft tissue complications (Chaudhary et al 2008). To minimise these complications, meticulous perioperative glycaemic control with an insulin sliding scale is essential as well as gentle soft tissue handling and robust fixation (Mehta et al 2014).

Later complications include malunion, non-union, secondary displacement and metalwork failure (Mehta et al 2014). Furthermore, patients are at risk of developing chronic pain, muscle atrophy and a stiff/swollen joint (Donken et al 2012). A long-term follow-up study reported that 63% of surgically treated ankle fracture patients had stiffness, 45% had ankle swelling, 50% experienced pain, and 38% had not returned to previous sporting levels, even five years after injury (Shah et al 2007). One of the most common long-term complications is post-traumatic osteoarthritis (PTOA). Advanced PTOA has been reported to affect 36.3% of surgically treated malleolar fracture patients at a mean of 18 years follow-up (Lübbeke et al 2012). It is reported that a lateral talar displacement of 1mm can reduce the contact area between talus and tibia by approximately 42%, which increases peak forces and cartilage wear and tear (Ramsey & Hamilton 1976). As surgical treatment of displaced fractures is more likely to restore normal anatomy, one may expect it to result in lower rates of PTOA. However, a Cochrane review comparing surgical and conservative treatment for ankle fractures concluded that there is insufficient evidence to definitively say which approach produces superior long‐term outcomes (Donken et al 2012).

Ankle fractures may also have a significant psychosocial impact. A recent study assessed the experiences of 10 ankle fracture patients using semi-structured interviews between 19 and 23 weeks following injury (McKeown et al 2020). These patients complained of a loss of independence, difficulties with activities of daily living, sleep disturbances, fatigue, depression and anxiety. These were attributable to factors such as reduced mobility, pain, skin issues and loss of strength and muscle bulk (McKeown et al 2020). Although this study included only a small number of patients and reported qualitative outcomes at a single point in time, it demonstrates the need for perioperative practitioners to take into account psychosocial factors when treating these individuals. Indeed, patients with ankle fractures may require psychological rehabilitation and social support in order to recover as quickly as possible (Mittly et al 2016). An improved understanding of these psychosocial factors will allow development of patient-tailored management plans.

### Weight-bearing after surgery

Traditionally, the postoperative advice after ORIF has been to remain non-weight-bearing for six to eight weeks (King et al 2020). However, more recent evidence suggests that early mobilisation before this time period may accelerate recovery (Smeeing et al 2015). A number of multi-centred randomised controlled trials have studied this issue. In a study of 115 adult patients, the unprotected weight-bearing group had a significantly higher ankle function score than the protected weight-bearing and non-weight-bearing groups at six weeks (Smeeing et al 2020). Moreover, the unprotected weight-bearing group had a significantly earlier return to work and sports with no difference in the complication rates. Lorente et al (2020) reported similar findings in 70 elderly patients over a longer follow-up period. Specifically, this group found that early weight-bearing patients scored higher than non-weight-bearing patients using two standardised measures of quality of life (SF-12 and Barthel Index) at six to eight weeks, one year and two years of follow-up (Lorente et al 2020).

There is therefore an emerging evidence base in favour of weight-bearing after ankle fracture. Indeed, current guidelines recommend that patients bear weight as tolerated in a splint/cast post-surgery, except where the stability of fixation is uncertain or there are co-morbidities such as peripheral neuropathy (BOA 2016). NICE also advises immediate, unrestricted weight-bearing as tolerated for patients with stable fractures (NICE 2016a).

## Virtual fracture clinics

Virtual fracture clinics (VFCs) are amongst the latest recommendations for research published by NICE (NICE 2016a). According to the BOAST guidelines, ‘following acute traumatic orthopaedic injury, patients should be seen in a new fracture clinic within 72 hours of presentation’ (BOA 2013). VFCs may help to meet this goal in the context of an overstretched, and financially limited traditional fracture clinic model (McKirdy & Imbuldeniya 2017). The use of VFCs has been shown to significantly decrease non-attendees and the number of days to first orthopaedic review as well as significantly increase the number of patients reviewed within 72 hours (McKirdy & Imbuldeniya 2017). Furthermore, VFCs saved a local Clinical Commissioning Group £67,385.67 in their first year of use, demonstrating clinical and cost-effectiveness (McKirdy & Imbuldeniya 2017). One concern though is that VFCs compromise the patient–doctor relationship. This has recently been challenged by high patient satisfaction rates, with 94% of patients rating the service as good or excellent, and 97% saying that they were likely or extremely likely to recommend it to others (Hawarden et al 2018).

## Conclusions

Ankle fractures are complex orthopaedic injuries associated with significant morbidity and mortality, especially in the perioperative period. It is therefore important to be conscious of the key perioperative factors that influence the care of these patients.

## Key phrases

1. The ankle is a complex region and many different injuries can occur.
2. Ankle fractures are associated with significant morbidity and occasionally mortality.
3. It is important to be aware of the physical and psychosocial complications of ankles fractures.
4. Early weight-bearing may improve rehabilitation in stable ankle fractures.

## References

[bibr1-1750458920969029] Bachmann LM Kolb E Koller MT Steurer J Ter Riet G 2003 Accuracy of Ottawa ankle rules to exclude fractures of the ankle and mid-foot: systematic review **Br Med J** 326 (7386) 417–419 12595378 10.1136/bmj.326.7386.417 PMC149439

[bibr2-1750458920969029] Bartoníček J Rammelt S Tuček M 2017 Posterior malleolar fractures: changing concepts and recent developments **Foot Ankle Clin** 22 (1) 125–145 28167058 10.1016/j.fcl.2016.09.009

[bibr3-1750458920969029] BOA 2013 Fracture clinic services Available at https://www.boa.ac.uk/uploads/assets/7ded8f00-987e-42d5-a389e739b1e03b47/ec9d4564-4fa7-4d08-aef4efc3cede7d53/fracture%20clinic%20services.pdf?fbclid=IwAR3DGE2YDtl6nmPDKwwwTwu6RTqH75DbZOHluOczjSijcF3gIMJ4u9khdZY [accessed March 2020]

[bibr4-1750458920969029] BOA 2016 The management of ankle fractures Available at https://www.boa.ac.uk/resources/boast-12-pdf.html [accessed March 2020]

[bibr5-1750458920969029] BOA & BAPRAS 2017 Open fractures Available at https://www.boa.ac.uk/uploads/assets/3b91ad0a-9081-4253-92f7d90e8df0fb2c/29bf80f11cb6-46b7-afc761119341447f/open%20fractures.pdf [accessed March 2020].

[bibr6-1750458920969029] Briet JP Hietbrink F Smeeing DP Dijkgraaf MGW Verleisdonk EJ Houwert RM 2019 Ankle fracture classification: an innovative system for describing ankle fractures **J Foot Ankle Surg** 58 (3) 492–496 30795890 10.1053/j.jfas.2018.09.028

[bibr7-1750458920969029] Bugler KE Clement ND Duckworth AD White TO McQueen MM Court-Brown CM 2015 Open ankle fractures: who gets them and why? **Arch Othop Trauma Surg** 135 (3) 297–303 10.1007/s00402-014-2140-3 25596941

[bibr8-1750458920969029] Chaudhary SB Liporace FA Gandi A Donley BG Pinzur MS Lin SS 2008 Complications of ankle fracture in patients with diabetes **J Am Acad Orthop Surg** 16 (3) 159–170 18316714 10.5435/00124635-200803000-00007

[bibr10-1750458920969029] Donken CC Al-Khateeb H Verhofstad MH Van Laarhoven CJ 2012 Surgical versus conservative interventions for treating ankle fractures in adults **Cochrane Database Syst Rev** (8) CD008470 22895975 10.1002/14651858.CD008470.pub2 PMC12145951

[bibr11-1750458920969029] Elsoe R Ostgaard SE Larsen P 2018 Population-based epidemiology of 9767 ankle fractures **Foot Ankle Surg** 24 (1) 34–39 29413771 10.1016/j.fas.2016.11.002

[bibr12-1750458920969029] Gandham S Millward G Molloy A Mason L 2020 Posterior malleolar fractures: a CT guided incision analysis **The Foot** 43 101662 32086138 10.1016/j.foot.2019.101662

[bibr13-1750458920969029] Haddix KP Clement RC III Tennant JN Ostrum RF 2018 Complications following operatively treated ankle fractures in insulin- and non-insulin-dependent diabetic patients **Foot Ankle Spec** 11 (3) 206–216 28617050 10.1177/1938640017714867

[bibr501-1750458920969029] Hawarden D, Boyle M, Robinson A, Alqubaisi M, Pillai A 2018 Virtual fracture clinic in the management of foot and ankle fractures ** *Res Rev Insights* ** 2 (4) 1–4

[bibr14-1750458920969029] Hsu RY Lee Y Hayda R DiGiovanni CW Mor V Bariteau JT 2015 Morbidity and mortality associated with geriatric ankle fractures **J Bone Joint Surg Am** 97 (21) 1748–1755 26537162 10.2106/JBJS.O.00095

[bibr15-1750458920969029] Jain S Haughton BA Brew C 2014 Intramedullary fixation of distal fibular fractures: a systematic review of clinical and funcitonal outcomes **J Orthop Traumatol** 15 (4) 245–254 25304004 10.1007/s10195-014-0320-0 PMC4244552

[bibr16-1750458920969029] Jennison T Brinsden M 2019 Fracture admission trends in England over a ten-year periods **Ann R Coll Surg Eng** 101 (3) 208–214 10.1308/rcsann.2019.0002 PMC6400910 30698459

[bibr17-1750458920969029] King CM Doyle MD Castellucci-Garza FM Nguyentat A Collman DR Schuberth JM 2020 Early protected weightbearing after open reduction internal fixation of ankle fractures with trans-syndesmotic screws **J Foot Ankle Surg** S1067-2516 (20) 30004–1 10.1053/j.jfas.2020.01.003 32057623

[bibr18-1750458920969029] Lampridis V Gougoulias N Sakellariou A 2018 Stability in ankle fractures: diagnosis and treatment **EFORT Open Rev** 3 (5) 294–303 29951269 10.1302/2058-5241.3.170057 PMC5994620

[bibr19-1750458920969029] Lauge-hansen N 1954 Fractures of the ankle. III. Genetic roentgenologic diagnosis of fractures of the ankle **Am J Roentgenol Radium Ther Nucl Med** 71 (3) 456–471 13124631

[bibr20-1750458920969029] Leung KH Fang CXS Lau TW Leung FKL 2016 Preoperative radiography versus computed tomography for surgical planning for ankle fractures **J Orthop Surg (Hong Kong)** 24 (2) 158–162 27574254 10.1177/1602400207

[bibr21-1750458920969029] Lorente A Palacios P Lorente R Mariscal G Barrios C Gandía A 2020 Orthopedic treatment and early weight-bearing for bimalleolar ankle fractures in elderly patients: quality of life and complications **Injury** 51 (2) 548–553 31767374 10.1016/j.injury.2019.11.028

[bibr22-1750458920969029] Lübbeke A Salvo D Stern R Hoffmeyer P Holzer N Assal M 2012 Risk factors for post-traumatic osteoarthritis of the ankle: an eighteen year follow-up study **Int Orthop** 36 (7) 1403–1410 22249843 10.1007/s00264-011-1472-7 PMC3385887

[bibr23-1750458920969029] Macera A Christian C Sirleo L Innocenti M 2018 Postoperative complications and reoperation rates following open reduction and internal fixation of ankle fracture **Joints** 6 (2) 110–115 30051108 10.1055/s-0038-1653949 PMC6059857

[bibr24-1750458920969029] McKeon JMM Hoch MC 2019 The ankle-joint complex: a kinesiologic approach to lateral ankle sprains **J Athl Train** 54 (6) 589–602 31184957 10.4085/1062-6050-472-17 PMC6602390

[bibr25-1750458920969029] McKeown R Kearney RS Liew ZH Ellard DR 2020 Patient experiences of an ankle fracture and the most important factors in their recovery: a qualitative interview study **BMJ Open** 10 (2) e033539 10.1136/bmjopen-2019-033539 PMC7044932 32024789

[bibr500-1750458920969029] McKirdy A, Imbuldeniya AM 2017 The clinical and cost effectiveness of a virtual fracture clinic service: An interrupted time series analysis and before-and-after comparison ** *Bone Joint Res* ** 6 (5) 259–269 10.1302/2046-3758.65.BJR-2017-0330.R1 PMC5457647 28473333

[bibr26-1750458920969029] Mehta S Rees K Cutler L Mangwani J 2014 Understanding risks and complications in the management of ankle fractures **Indian J Orthop** 48 (5) 445–452 25298549 10.4103/0019-5413.139829 PMC4175856

[bibr27-1750458920969029] Mittly V Németh Z Berényi K Mintál T 2016 Mind does matter: the psychological effect of ankle injury in sport **J Psychol Psychother** 6 (4) 1–7

[bibr28-1750458920969029] Moore KL Dalley AF Agur AMR 2014 **Clinically Oriented Anatomy** 7th Edition Philadelphia, PA, Lippincott Williams & Wilkins

[bibr29-1750458920969029] Mordecai S Al-Hadithy N 2011 Management of ankle fractures **BMJ** 343 d5204. https://www.bmj.com/content/343/bmj.d5204.abstract?fbclid=IwAR2v_IW-tj2cbddgDHzC8dfwRjh7dFLa6kqxm1jinlalyaEjwCDvrkiMlKI 22039272 10.1136/bmj.d5204

[bibr30-1750458920969029] NICE 2016a Fractures (non-complex): assessment and management Available at https://www.nice.org.uk/guidance/ng38 [accessed March 2020].

[bibr31-1750458920969029] NICE 2016b Fractures (complex): assessment and management Available at https://www.nice.org.uk/guidance/ng37/chapter/Recommendations [accessed March 2020].

[bibr32-1750458920969029] Odak S Ahluwalia R Unnikrishnan P Hennessy M Platt S 2016 Management of posterior malleolar fractures: a systematic review **J Foot Ankle Surg** 55 (1) 140–145 26100091 10.1053/j.jfas.2015.04.001

[bibr34-1750458920969029] Ramsey PL Hamilton W 1976 Changes in tibiotalar area of contact caused by lateral talar shift **J Bone Joint Surg Am** 58 (3) 356–357 1262367

[bibr35-1750458920969029] Sawant Y Sanghvi D 2018 Magnetic resonance imaging of ankle ligaments: a pictorial essay ** *Indian* ** **J Radiol Imaging** 28 (4) 419–426 10.4103/ijri.IJRI_77_16 PMC6319113 30662202

[bibr36-1750458920969029] Shah KN Johnson JP O’Donnell SW Gil JA Born CT Hayda RA 2019 External fixation in the emergency department for pilon and unstable ankle fractures **J Am Acad Orthop Surg** 27 (12) e577–e584 30394911 10.5435/JAAOS-D-18-00080

[bibr37-1750458920969029] Shah NH Sundaram RO Velusamy A Braithwaite IJ 2007 Five-year functional outcome analysis of ankle fracture fixation **Injury** 38 (11) 1308–1312 17888434 10.1016/j.injury.2007.06.002

[bibr38-1750458920969029] Slimmon D Brukner P 2010 Sports ankle injuries – assessment and management **Aust Fam Physician** 39 (1–2) 18–22 20369129

[bibr39-1750458920969029] Smeeing DPJ Houwert RM Briet JP , et al 2015 Weight-bearing and mobilization in the postoperative care of ankle fractures: a systematic review and meta-analysis of randomized controlled trials and cohort studies **PLOS ONE** 10 (2) e0118320 25695796 10.1371/journal.pone.0118320 PMC4335061

[bibr40-1750458920969029] Smeeing DPJ Houwert RM Briet JP , et al 2020 Weight-bearing or non-weight-bearing after surgical treatment of ankle fractures: a multicenter randomized controlled trial **Eur J Trauma Emerg S** 46 (1) 121–130 10.1007/s00068-018-1016-6 PMC7026225 30251154

[bibr41-1750458920969029] Solan M Sakellariou A 2017 Posterior malleolus fractures: worth fixing **Bone Joint J** 99-B (11) 1413–1419 10.1302/0301-620X.99B11.BJJ-2017-1072 29092978

[bibr42-1750458920969029] Srinivasan CMS Moran CG 2001 Internal fixation of ankle fractures in the very elderly **Injury** 32 (7) 559–563 11524089 10.1016/s0020-1383(01)00034-1

[bibr43-1750458920969029] Stiell IG Greenberg GH McKnight RD Nair RC McDowell I Worthington JR 1992 A study to develop clinical decision rules for the use of radiography in acute ankle injuries **Ann Emerg Med** 21 (4) 384–390 1554175 10.1016/s0196-0644(05)82656-3

[bibr44-1750458920969029] Stoffel K Wysocki D Baddour E Nicholls R Yates P 2009 Comparison of two intraoperative assessment methods for injuries to the ankle syndesmosis. A cadaveric study **J Bone Joint Surg Am** 91 (11) 2646–2652 19884439 10.2106/JBJS.G.01537

[bibr45-1750458920969029] Vangsness CT Carter V Newton E Hunt T Kerr R 1994 Radiographic diagnosis of ankle fractures: are three views necessary? **Foot Ankle Int** 15 (4) 172–174 7951949 10.1177/107110079401500403

[bibr46-1750458920969029] Zalavras CG Christensen T Rigopoulos N Holtom P Patzakis MJ 2009 Infection following operative treatment of ankle fractures **Clin Orthop Relat Res** 467 (7) 1715–1720 19225850 10.1007/s11999-009-0743-8 PMC2690750

